# Bowman-Birk Protease Inhibitor from *Vigna unguiculata* Seeds Enhances the Action of Bradykinin-Related Peptides

**DOI:** 10.3390/molecules191117536

**Published:** 2014-10-30

**Authors:** Alice da Cunha M. Álvares, Elisabeth Ferroni Schwartz, Nathalia Oda Amaral, Neidiane Rosa Trindade, Gustavo Rodrigues Pedrino, Luciano Paulino Silva, Sonia Maria de Freitas

**Affiliations:** 1 Laboratório de Biofísica, Instituto de Biologia, Departamento de Biologia Celular, Universidade de Brasília-UnB, Quadra 604, Asa Norte, Bloco J 1° andar, Brasília, DF 70910-900, Brazil; E-Mail: pharmalice@gmail.com; 2 Laboratório de Toxinologia, Departamento de Ciências Fisiológicas, Universidade de Brasília-UnB, Quadra 604, Asa Norte, Bloco J Térreo, Brasília, DF 70910-900, Brazil; E-Mail: efschwa@unb.br; 3 Center of Neuroscience and Cardiovascular Physiology, Department of Physiological Sciences, Biological Sciences Institute, Federal University of Goiás, Estrada do Campus, Goiânia, GO 74690-900, Brazil; E-Mails: oda_ufg@posgrad.ufg.br (N.O.A.); neidiane@posgrad.ufg.br (N.R.T.); pedrino@ufg.br (G.R.P.); 4 Embrapa Recursos Genéticos e Biotecnologia, Laboratório de Espectrometria de Massa, PBI, Parque Estação Biológica, Final W5 Norte, Asa Norte, Brasília, DF 70770-917, Brazil; E-Mail: luciano.paulino@embrapa.br

**Keywords:** arterial blood pressure, bradykinin, Bowman-Birk inhibitor, circular dichroism, dynamic light scattering, fluorescence spectroscopy

## Abstract

The hydrolysis of bradykinin (Bk) by different classes of proteases in plasma and tissues leads to a decrease in its half-life. Here, Bk actions on smooth muscle and *in vivo* cardiovascular assays in association with a protease inhibitor, Black eyed-pea trypsin and chymotrypsin inhibitor (BTCI) and also under the effect of trypsin and chymotrypsin were evaluated. Two synthetic Bk-related peptides, Bk_1_ and Bk_2_, were used to investigate the importance of additional C-terminal amino acid residues on serine protease activity. BTCI forms complexes with Bk and analogues at pH 5.0, 7.4 and 9.0, presenting binding constants ranging from 10^3^ to 10^4^ M^−1^. Formation of BTCI-Bk complexes is probably driven by hydrophobic forces, coupled with slight conformational changes in BTCI. *In vitro* assays using guinea pig (*Cavia porcellus*) ileum showed that Bk retains the ability to induce smooth muscle contraction in the presence of BTCI. Moreover, no alteration in the inhibitory activity of BTCI in complex with Bk and analogous was observed. When the BTCI and BTCI-Bk complexes were tested *in vivo*, a decrease of vascular resistance and consequent hypotension and potentiating renal and aortic vasodilatation induced by Bk and Bk_2_ infusions was observed. These results indicate that BTCI-Bk complexes may be a reliable strategy to act as a carrier and protective approach for Bk-related peptides against plasma serine proteases cleavage, leading to an increase in their half-life. These findings also indicate that BTCI could remain stable in some tissues to inhibit chymotrypsin or trypsin-like enzymes that cleave and inactivate bradykinin *in situ*.

## 1. Introduction

Bradykinin (Bk) is an endogenous biologically active peptide with nine amino acid residues (Arg_1_-Pro_2_-Pro_3_-Gly_4_-Phe_5_-Ser_6_-Pro_7_-Phe_8_-Arg_9_), which is physiologically cleaved from kininogen by serine proteases, such as kallikreins. Overall, the pharmacological activity of kinins is associated with many physiological and pathological processes and is also related to the renin-angiotensin system, blood coagulation and complementary pathways [1,2]. Non-mammalian bradykinin-related peptides, including those from amphibian, show primary structure differences, which are consistent with functional specificities. Thus, there is an increasing interest on the investigation of such peptides in comparison to the classic bradykinin.

Bradykinin and related kinins act in the constriction and vasodilatation of smooth muscle, as well as promoting arterial hypotension acting on two types of receptors, B_1_ and B_2_. Whereas B_1_ receptors are barely expressed in normal tissues, being up-regulated after lesion and inflammation [3], B_2_ receptors are responsible for most of the physiological effects of the kinins, including arterial vasodilatation, and peripheral smooth muscle contraction (stomach, colon, urinary bladder, uterus, and the ileum) [4,5]. *In vivo* hypotensive action results from vasodilatation by stimulation of endothelial B_2_ receptors in arteries and arterioles, as well as from renal action in the natriuresis process [6,7,8,9]. Its hypotensive effect is decreased in plasma and tissues in the presence of kininases, such as the Angiotensin Converting Enzyme (ACE) [10,11,12], metalloproteases [11] and chymotrypsin. In order to increase the half-life of Bk *in vitro* or in plasma and tissues, an association with protease inhibitors could be used as a rational strategy to prevent Bk enzymatic hydrolysis. Studies on the effects of ACE inhibitors showed that these molecules attenuate the progression of arteriosclerosis and the occurrence of cardiovascular events in humans [13]. In this context, protease inhibitors have been considered as one of the main pharmacological targets for cardiovascular treatment.

The huge interest in protease inhibitors has focused on natural inhibitors from different sources, particularly leguminous plants that are capable of regulating a number of relevant biological processes. These inhibitors belong to the well-characterized Bowman-Birk Inhibitor (BBI) and Kunitz-type inhibitor families [14]. In particular, BBI have been the most widely investigated molecules from the physicochemical, structural and functional points of view. BBIs play an important role in plant defense mechanisms against pathogens [15,16,17] and in various biological processes and therapeutic applications. They are involved in the inhibition of intracellular protein hydrolysis, in transcription and cell cycle, and cell invasion [18,19]. In addition, these inhibitors have also been described as anticarcinogenic agents acting on the prevention and suppression of cancer in several organs and tissues *in vitro* and *in vivo* [20,21,22,23,24,25].

The Black-eyed pea Trypsin and Chymotrypsin Inhibitor (BTCI) is a member of the BBI family and was isolated from *Vigna unguiculata* seeds. This inhibitor is a stable globular protein consisting of 83 amino acid residues and seven disulfide bonds [26]. It presents two different reactive sites that interact simultaneously and independently with trypsin and chymotrypsin by forming binary and ternary stable complexes [27,28,29]. Furthermore, BTCI was characterized as the first member of the BBI family that elicited effects on renal function in rats [30]. BTCI enhanced guanylin-induced natriuresis response leading to an increase in urinary flow, in fractional excretion of Na^+^ and K^+^, in perfusion pressure, in glomerular filtration rate and allowing osmolar clearance. BTCI probably enhanced the natriuretic effects of this peptide through inhibition of its degradation by proteases present in this urinary system.

To date, no studies about the association of serine protease inhibitors, specifically those belonging to the BBI family and biologically active Bk, have been reported. However, studies regarding protease inhibitors from several sources generally focus on their effects on kallikreins by inhibiting the release of Bk from kininogen [1].

In the present study, we report the association of BTCI with classical bradykinin and its analogues and the protective action of BTCI against proteolytic degradation of Bk, as well as the effect of BTCI on their *in vitro* and *in vivo* hypotensive activities. The structural features of BTCI and its inhibitory activity, in the presence of classical Bk and two Bk analogues, were also investigated. The *in vitro* and *in vivo* experiments of Bk-related peptides in the presence or absence of BTCI *in vivo* were performed to assess smooth muscle contraction effects and cardiovascular responses induced by intravenous administration, respectively.

## 2. Results and Discussion

### 2.1. Synthesis and Purification of Bk and Bk-Related Peptides

**Figure 1:**
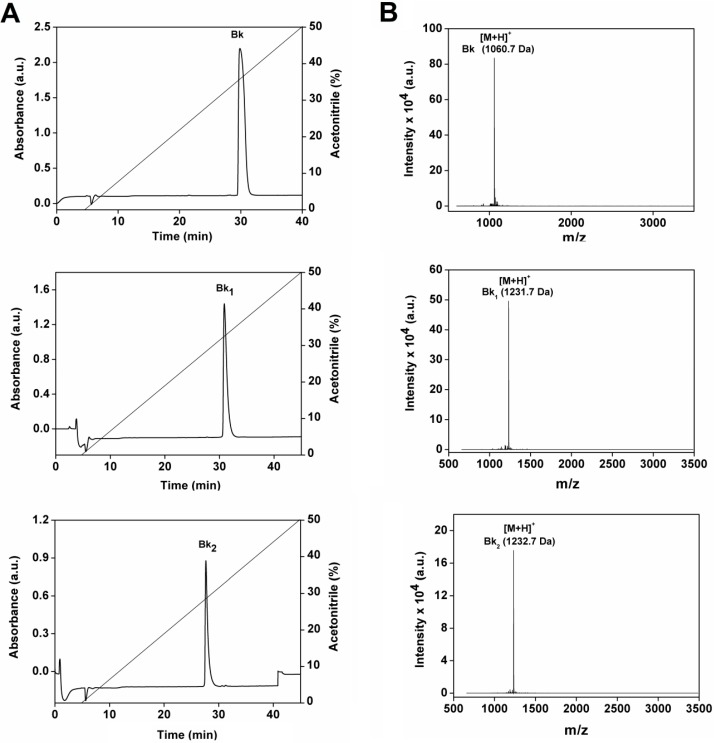
Purification of the synthetic peptides Bk, Bk_1_ and Bk_2_. (**A**) Reverse-phase chromatography C_18_ Vydac 218 TP 510 column using a linear gradient (5%–95%) of acetonitrile (ACN); (**B**) MALDI-TOF mass spectrometry analysis of synthetic peptides Bk, Bk_1_ and Bk_2_ identified by the legend.

Bk [Arg^1^-Pro^2^-Pro^3^-Gly^4^-Phe^5^-Ser^6^-Pro^7^-Phe^8^-Arg^9^], Bk_1_ [Val]^1^[Thr]^6^-bradykinyl-Val-Asp and Bk_2_ [Val]^1^[Thr]^6^-bradykinyl-Gln-Ser (Table 1) were chemically synthesized and purified by semi-preparative high performance liquid chromatography (HPLC), as shown in Figure 1A. All peptides are relatively hydrophobic, as indicated by hydrophobic moment values shown in Table 1. The purity and molecular mass of three Bk-related peptides (Bk 1060.7 Da; Bk_1_ 1231.7 Da and Bk_2_ 1232.7 Da) were confirmed by matrix-assisted laser desorption/ionization time of flight mass spectrometry (MALDI–TOF MS) analyses, as indicated by a single spectrum obtained for each peptide (Figure 1B). The pretreatment *in vitro* of Bk_1_ and Bk_2_ with trypsin release the active Bk fragment as assessed by HPLC and mass spectrometry (data not shown).

**Table 1:** Amino acid sequences of the bradykinin and analogues.

		1	2	3	4	5	6	7	8	9	10	11	M.M. (Da)	I.P.	H.M.
Bk	Bradykinin	R	P	P	G	F	S	P	F	R	---	---	1060.7	12.0	1.195
Bk_1_	Val,[T]^6^-bradykinyl-Val,Asp	V	P	P	G	F	T	P	F	R	V	D	1231.7	5.8	1.450
Bk_2_	Val,[T]^6^-bradykinyl-Glu,Ser	V	P	P	G	F	T	P	F	R	Q	S	1232.7	9.7	2.443

Notes: M.M. is the Molecular Mass; I.P. is the Isoelectric Point; H.M. is the Hydrophobic Moment.

### 2.2. BTCI and Bk Complex Formation Investigated by Dynamic Light Scattering (DLS)

**Figure 2:**
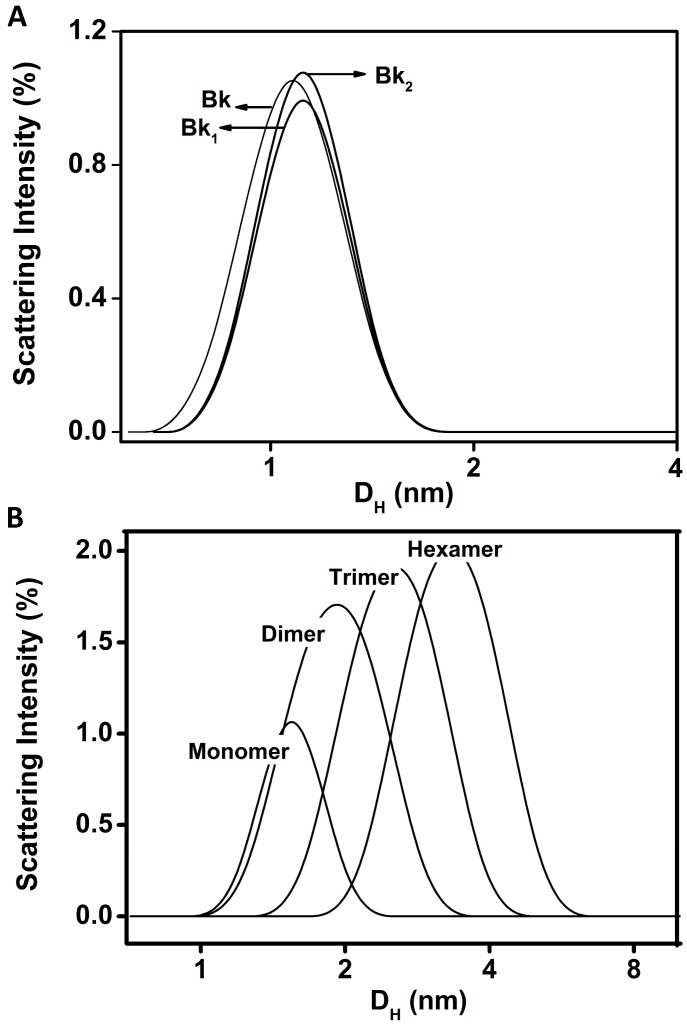

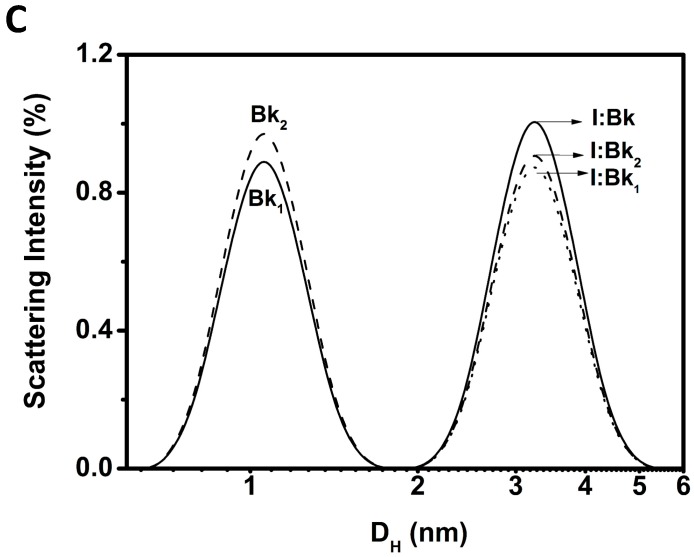
DLS size distribution (hydrodynamic diameter, D_H_) of Bk and its analogues (**A**), BTCI (**B**) and the BTCI-Bk-related peptide complexes (**C**).

The hydrodynamic diameters of 1.0–1.2 nm indicate that Bk and analogues appear as monomers at 25 µM, pH 7.4 and 37 °C (Figure 2A). The recorded polydispersity was lower than 0.15, which is compatible with a monodisperse system and purity of Bk and analogues in solution. This result also indicates that peptides do not present tendency to aggregate [31]. Nevertheless, BTCI appeared as a monomer at low concentration (25 µM), with approximately 1.6 nm hydrodynamic diameter, whereas at 100 µM it self-assembled into dimer, trimer, and hexamer (Figure 2B), with hydrodynamic diameter ranging from 1.9 to 3.5 nm. These results corroborated those previously reported, indicating the self-association of BTCI in different multimeric states, when in solution [32] or air-dried [33]. These multimeric states contributed to the observed polydispersity, which was greater than 0.3, compatible with oligomeric states of this molecule in solution. This process is favored by the hydrophobic surface of BTCI, which consists of several hydrophobic amino acid residues exposed to the solvent [28,29,34].

Bk, Bk_1_ and Bk_2_ presented a hydrodynamic diameter (D_H_) of 1.0–1.2 nm (Figure 2A) with polydispersity lower than 0.15 (monodisperse solution). BTCI is observed as a monomer at low concentrations (25 μM), and as a dimer, trimer and hexamer, in higher concentrations (100 μM) (Figure 2B), with a podispersity above 0.3. The hydrodynamic diameter of BTCI in the presence of Bk, Bk_1_ and Bk_2_ increased from 1.7 nm to 3.3 nm (Figure 2C). In addition, the BTCI-Bk complex formation was also indicated by the monodispersivity feature (polydispersity of 0.107) and the hydrodynamic diameter of about 3.3 nm, compatible with the dimensions of both associated molecules (Figure 2C). The monodispersity of Bk-BTCI complex and the moderate polidispersity of Bk_1_ and Bk_2_ complexed with BTCI indicates that these peptides bind to BTCI in different ways.

### 2.3. Structural Analysis of BTCI and BTCI-Bk-Related Peptide Complexes by Fluorescence Spectroscopy

**Figure 3:**
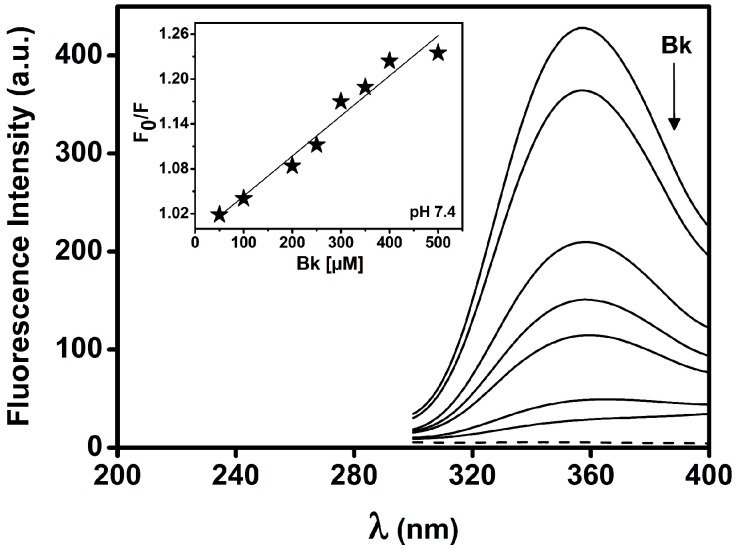
Fluorescence quenching of BTCI in complex with Bk at pH 7.4. Increasing concentrations of Bk (25 to 500 µM), indicated by arrow, lead to progressive reduction of Trp^82^ fluorescence intensity of BTCI. The inset shows a Stern-Volmer plot to obtain the K_sv_ values.

BTCI (25 µM) in complex with Bk and analogues were evaluated by fluorescence quenching within the concentration of peptides range of 25–500 µM. Addition of increasing concentrations of peptides caused a progressive decrease in fluorescence intensity, as shown for Bk in Figure 3. According to Stern-Volmer adjustment, a linear correlation is noted in the fluorescence emission at 355 nm (Figure 3, inset), consistent with a quenching occurring due to the static process and protein-protein complex formation. The Stern-Volmer constants (K_sv_), obtained at pH 5.0, 7.4 and 9.0 are shown in Table 2.

**Figure 4:**
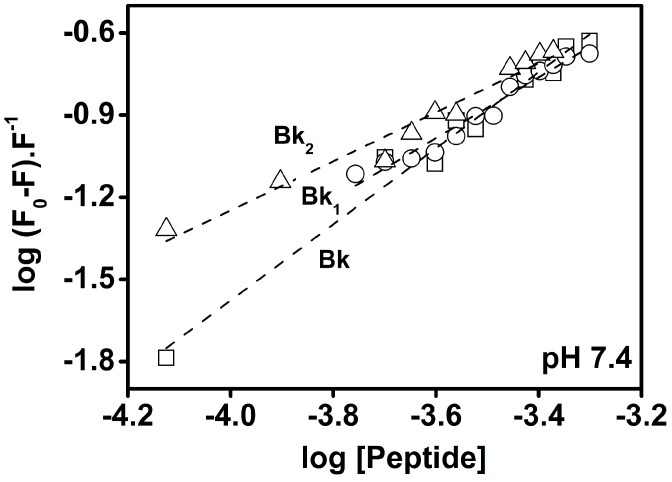
The plots of Log (F_0_−F/F) *vs.* Log [Peptide] for quenching of BTCI by Bk-related peptides, at pH 7.4.

The binding constant (K_b_) and the number of binding sites per BTCI molecule (*n*), of each Bk-related peptide association, as a function of pH, were calculated from fluorescence quenching at pH 5.0, 7.4 and 9.0. However, only the binding assay for Bk, analogues and BTCI at pH 7.4 is highlighted (Figure 4). The binding constants at different pHs (Table 3) were calculated assuming the equilibrium between free and bound small molecules that can bind independently to a set of equivalent sites in a single macromolecule. The high correlation coefficients and low standard deviations indicated that the results derived from Equation (3) (see methods) are satisfactory.

**Table 2:** Stern-Volmer quenching constants (K_SV_) of the BTCI in complex with Bk, Bk_1_ and Bk_2_ at different pHs.

Peptide	pH	K_SV_ (×10^2^ M^−1^)	R ^2a^	S.D. ^b^
	5.0	5.02	0.97	0.27
Bk	7.4	5.53	0.97	0.30
	9.0	4.68	0.99	0.15
	5.0	5.00	0.97	0.25
Bk_1_	7.4	4.82	0.97	0.26
	9.0	4.38	0.97	0.22
	5.0	4.25	0.99	0.11
Bk_2_	7.4	5.09	0.97	0.28
	9.0	3.84	0.98	0.17

Notes: ^a^ Correlation coefficient; ^b^ Standard deviation.

**Table 3:** Binding constants K_b_ and binding sites (*n*) of BTCI in complex with Bk, Bk_1_ and Bk_2_ at different pHs.

Peptide	pH	K_b_ (×10^3^ M^−1^)	*n* ^a^	R ^2b^	S.D. ^c^
	5.0	0.21	0.88	0.94	0.07
Bk	7.4	9.68	1.39	0.98	0.07
	9.0	0.71	1.06	0.98	0.04
	5.0	0.84	1.08	0.96	0.06
Bk_1_	7.4	1.02	1.11	0.96	0.06
	9.0	0.23	0.91	0.97	0.05
	5.0	0.59	1.04	0.98	0.04
Bk_2_	7.4	0.22	0.89	0.96	0.06
	9.0	1.81	1.23	0.98	0.06

Notes: ^a^ number of site; ^b^ correlation coefficient; ^c^ standard deviation.

### 2.4. Secondary Structure and Inhibitory Activity of BTCI in the Presence of Bk-related Peptides

**Figure 5:**
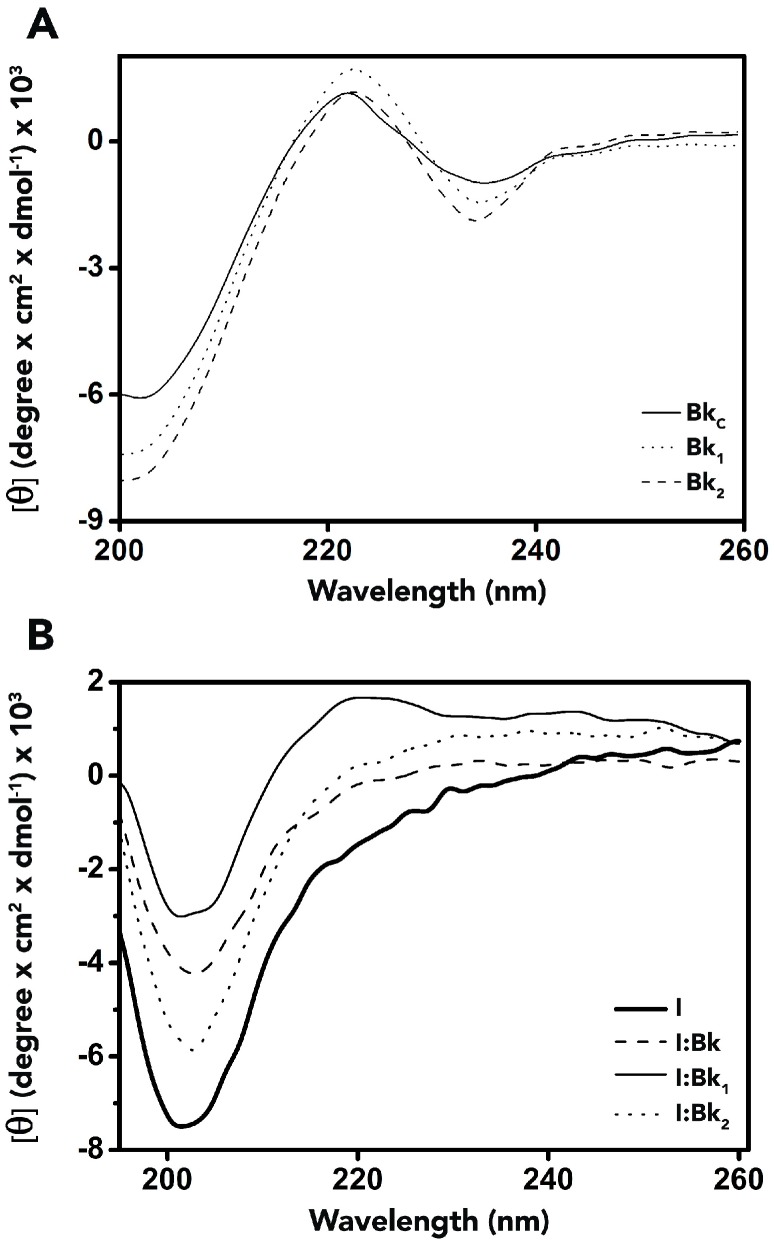
Far-UV Circular dichroism (CD) spectra of Bk-related peptides, BTCI and their complexes. (**A**). CD spectra of Bk, Bk_1_ and Bk_2_ show a negative dichroic band at 235 nm, a positive band at 221 nm and a negative band at 210 nm; (**B**). CD spectra of the complexes between Bk-related peptides and BTCI showing differences in the molar elipticity intensity of the bands.

The conformational changes of Bk and analogues, BTCI free and in complex with peptides at pH 7.4 were evaluated by circular dichroism (CD). Far-UV CD spectra of Bk and its analogues (Figure 5A) are similar. Two weak dichroic bands, a negative one at 235 nm and a positive one at 220 nm, and an intense negative band at 210 nm indicate the peptides are in an unordered structure. The significance of maximum bands around 220 nm and minima around 235 nm is unknown, but most of the biologically active Bk-related peptides showed this dichroic profile [35,36,37]. Far-UV CD spectra of BTCI and BTCI in the presence of the Bk-related peptides were also similar presenting dichroic bands around 200 nm. However, they presented a small difference in molar ellipticity intensity (Figure 5B), consistent with small changes in the secondary structure content of BTCI (Table 4).

**Figure 6:**
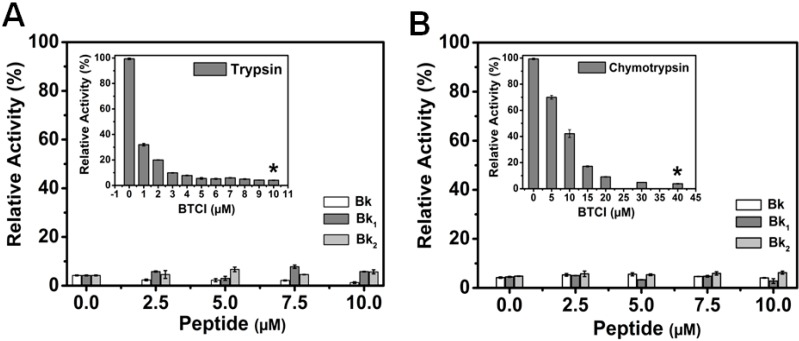
Trypsin and chymotrypsin activity in the presence of BTCI complexed with Bk and its analogues. (**A**) BTCI at 10 μM was set as the standard concentration to inhibit trypsin activity (inset); (**B**) BTCI at 40 μM was set as the standard concentration to inhibit chymotrypsin activity (inset). * *p* < 0.05 is compared with all other peptides at the same concentration.

The insets in Figure 6A,B show the inhibitory assays of BTCI against trypsin and chymotrypsin, respectively, as previously reported [38,39]. BTCI was highly active against bovine pancreatic trypsin at concentrations ranging from 0 to 10 µM, inhibiting about 100% of proteolytic activity at 10 µM (Figure 6A, inset). BTCI was also highly active against bovine chymotrypsin, inhibiting approximately 100% of its proteolytic activity at 40 µM (Figure 6B, inset).

The inhibitory activities of BTCI at concentrations in which it showed ~100% of protease inhibition (10 µM for trypsin and 40 µM for chymotrypsin) in the presence of increasing concentrations of Bk-related peptides (0–10 µM) were very similar (Figure 6A,B). Bk enhanced BTCI inhibition activity against trypsin and chymotrypsin, whereas Bk_1_ and Bk_2_ did not interfer in their activities.

**Table 4:** Secondary structure contents of the BTCI and BTCI-Bk complexes.

Secondary Structure (%)	BTCI	BTCI-Bk	BTCI-Bk_1_	BTCI-Bk_2_
α-helix	11.6	11.1	12.6	14.7
β-antiparallel	17.4	21.0	46.7	30.0
β-parallel	22.0	19.0	5.4	16.5
β-turn	22.1	23.2	21.9	21.4
Random coil	56.8	55.7	32.3	49.0

Notes: The secondary structures were calculated using the CDNN deconvolution software (Version 2.1, Bioinformatik.biochemtech.uni-halle.de/cdnn) [40].

### 2.5. Modulation of Smooth Muscle Contraction Action of Bk Mediated by BTCI

The action of Bk and its analogues on smooth muscle was evaluated when they were in complex with BTCI, using guinea pig ileum preparations, a well-characterized smooth muscle contraction assay, sensitive to Bk. It is worth mentioning that B_2_ receptors in rat and guinea pig tissues belong to a similar pharmacological entity [4], so the vasodilatation observed in rat aorta experiments is related to the guinea pig ileum contraction, mediated through the same B_2_ subtype receptor.

**Figure 7:**
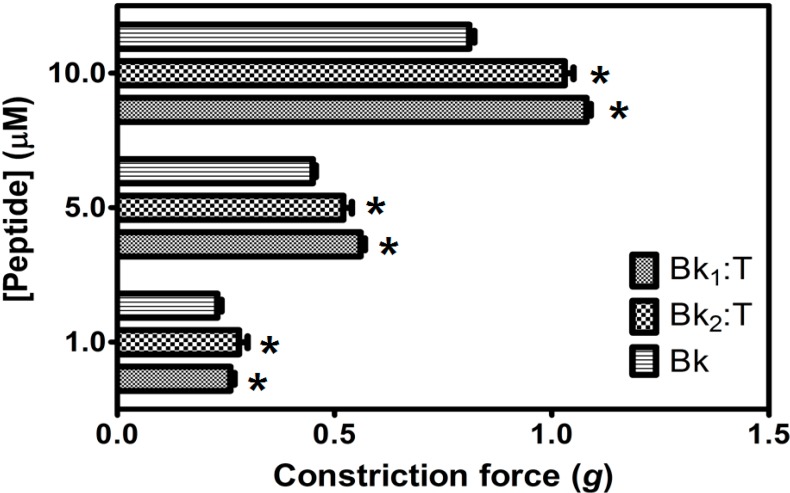
Smooth-muscle constriction force assay of Bk_1_ and Bk_2_ in different concentrations (1.0, 5.0 and 10.0 μM), in guinea pig ileum smooth-muscle. * *p* < 0.05 is compared with all other peptides at the same concentration.

**Figure 8:**
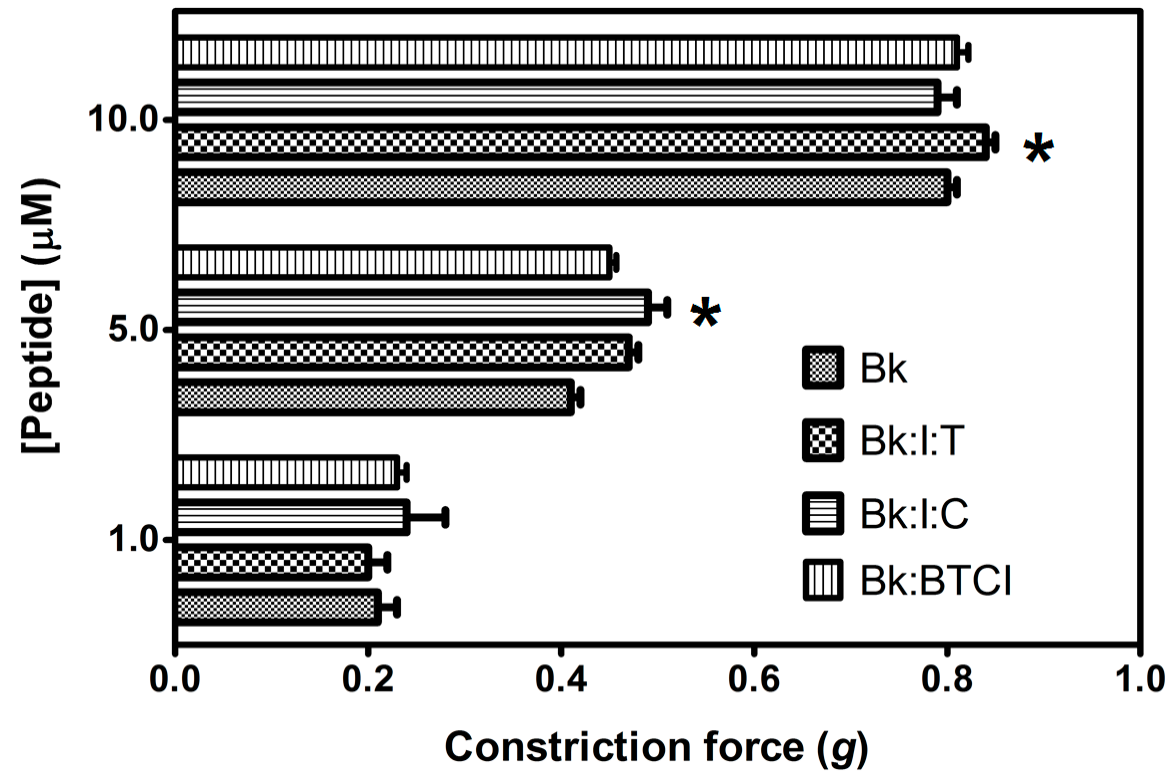
Smooth-muscle constriction assay of Bk in free state (Bk), complexed with BTCI (Bk:I), and complexed with trypsin (Bk:I:T) and chymotrypsin (Bk:I:C). Bk in different concentrations (1.0, 5.0 and 10.0 μM) was used to evaluate the induced contraction force in guinea pig ileum smooth-muscle * *p* < 0.05 is compared with all other peptides at the same concentration.

As shown in Figure 7 and Figure 8, different concentrations of Bk up to 10 µM were able to induce smooth muscle contraction in a concentration-responsive manner. However, Bk_1_ and Bk_2_ were unable to exert their contractive effects, suggesting that further structural processing for interaction with B_2_ receptors or a higher dose than that used in the present assay are necessary.

When pretreated with trypsin prior to application on guinea pig ileum, Bk_1_ and Bk_2_ were cleaved on Arg^9^-Val^10^ and Arg^9^-Gln^10^ and induced smooth muscle contraction, in a concentration-responsive manner (Figure 7), and at higher levels than Bk in free state, highlighting the importance of Arg^9^ for B_2_ receptor interaction. The action of Bk was evaluated in presence of BTCI and serine proteases to analysis if this inhibitor can influence the contraction on smooth muscle (Figure 8). Presence of BTCI slightly increased the smooth muscle contraction induced by Bk at 5.0 µM, and at 10.0 µM it was observed the same effect only when BTCI inhibited trypsin. From data present in Figure 8, we can observe that the effect of Bk was not affected by its association with BTCI, neither by the presence of serine proteases. Bk_1_ and Bk_2_ did not induce muscle contraction when pretreated with BTCI and serine proteases (data not shown). Contrary to *in vitro* results, the active fragments of Bk_1_ and Bk_2_ could be produced by the action of proteases *in vivo*. This could explain the reported *in vivo* hypotensive effects of such peptides.

### 2.6. Cardiovascular Effects of the Infusion of BTCI or Vehicle

**Figure 9:**
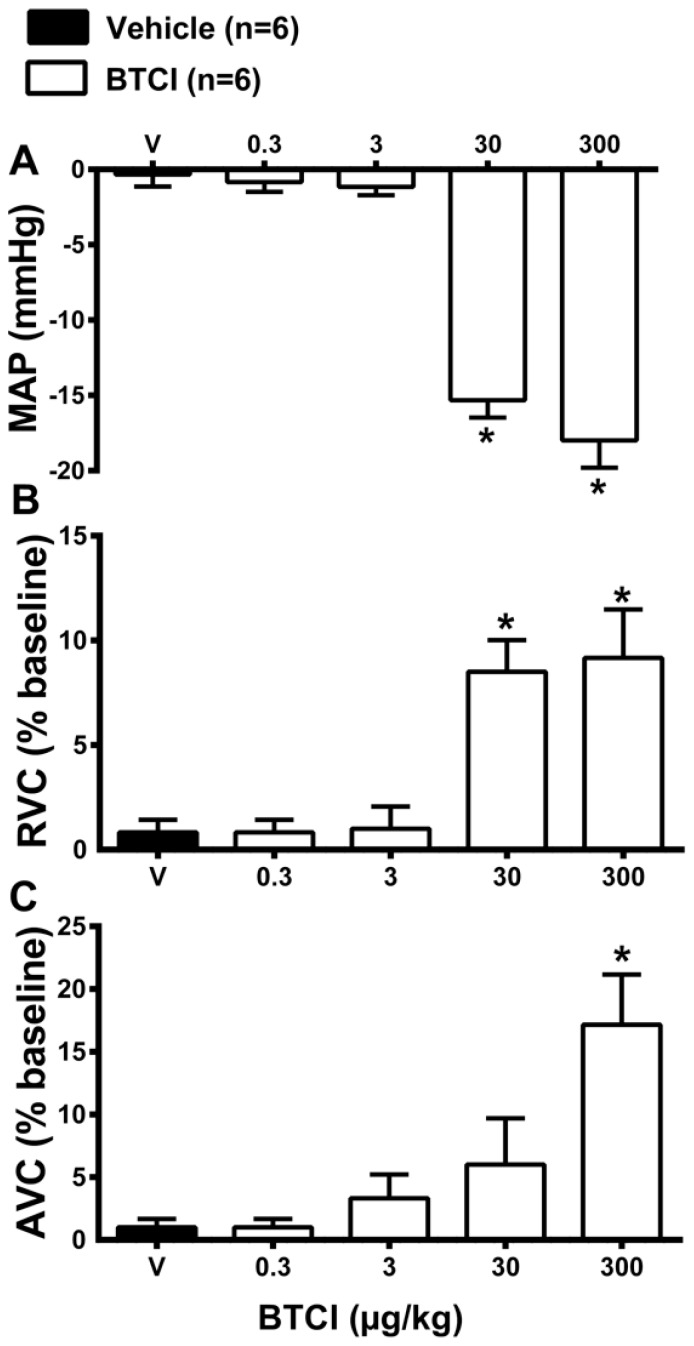
BTCI and hemodynamic effects. Cardiovascular responses induced by intravenous administration of BTCI (0.3, 3, 30 and 300 µg·kg^−1^ of b.wt, in 0.1 mL) or Vehicle (V; NaCl 0.9%), in anesthetized rats. (**A**) Mean arterial pressure (MAP); (**B**) Renal vascular conductance (RVC); (**C**) Aortic vascular conductance (AVC). The results are expressed as the mean ± SEM of 6 experiments. * *p* < 0.05 compared to V.

Preliminary experiments were performed to determine the dose of BTCI that does not induce cardiovascular changes. For this purpose, increasing doses of BTCI (0.3, 3, 30 and 300 µg·kg^−1^, i.v.) or vehicle (V; NaCl 0.9%) were infused. As expected, infusions of vehicle did not promote changes in mean arterial pressure (MAP; −0.3 ± 0.6 mmHg from the baseline value; Figure 9A), renal vascular conductance (RVC; 1 ± 0.5 bpm from the baseline value; Figure 9B) and aortic vascular conductance (AVC; 1.5% ± 0.7% from the baseline value; Figure 9C). BTCI infusions at the doses of 0.3 and 3 µg·kg^−1^ did not cause changes in MAP (−0.5 ± 0.7 and −1 ± 0.5 mmHg from the vehicle, respectively; Figure 9A), RVC (1 ± 0.2 and 1.2% ± 1% from the vehicle, respectively; Figure 9B) and AVC (1.5 ± 0.4 and 4% ± 1.4% from the vehicle, respectively; Figure 9C). However, high doses of BTCI, 30 and 300 µg·kg^−1^, decreased MAP (−15 ± 1.1 and −18 ± 1.8 mmHg from the baseline, respectively; Figure 9A), increased RVC (8 ± 1.6 and 9% ± 3.4% from the baseline, respectively; Figure 9B) and increased AVC (6 ± 2.1 and 17% ± 3.5% from the baseline, respectively; Figure 9C). After these results the dose of 3 µg·kg^−1^ was chosen to perform the next series of experiments.

### 2.7. Cardiovascular Effects of the Infusion of Bk and Bk-related Peptides in Association with BTCI or Vehicle

**Figure 10:**
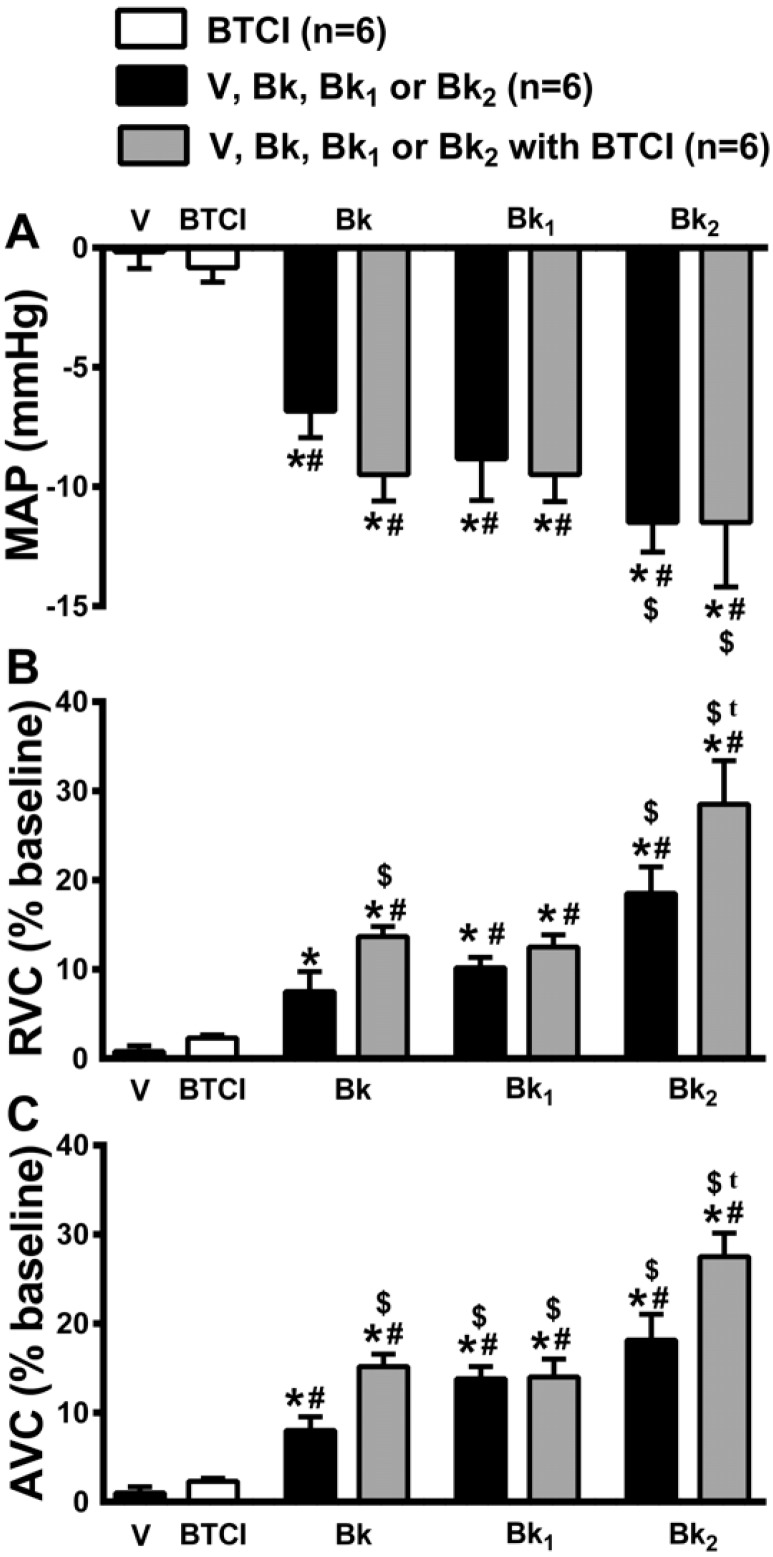
Bk and Bk related peptides and hemodynamic effects. Cardiovascular responses induced by intravenous administration of BTCI (3 µg·kg^−1^ of b.wt, in 0.1 mL), Vehicle (V; NaCl 0.9%), Bk, Bk_1_, Bk_2_ (120 µg·kg^−1^ of b.wt, in 0.1 mL, each) or Bk, Bk_1_ or Bk_2_ associated with BTCI or V in anesthetized rats. (**A**) Mean arterial pressure (MAP); (**B**) Renal vascular conductance (RVC); (**C**) Aortic vascular conductance (AVC). The results are expressed as the mean ± SEM of 6 experiments. * *p* < 0.05 compared to Vehicle; # Compared to BTCI, $ compared to Bk; t compared to Bk_2_.

Bk and its related peptides (Bk_1_ and Bk_2_) at the dose of 120 µg·kg^−1^ (i.v.) in association with BTCI (3 µg·kg^−1^, i.v.) or vehicle (NaCl 0.9%) were administrated intravenously to different groups of animals to evaluate the possible hemodynamic effects. MAP (−0.3 ± 0.6 mmHg from the baseline value; Figure 10A), RVC (1 ± 0.5 bpm from the baseline value; Figure 10B) and AVC (1.5% ± 0.7% from the baseline value; Figure 10C). On the contrary, intravenous administration of Bk and Bk-related peptides produced significant hypotension (Bk: −7 ± 1.2, Bk_1_: −9 ± 1.8 and Bk_2_: −12 ± 1.2 mmHg from the baseline value; Figure 10A), renal (Bk: 9 ± 2, Bk_1_: 10 ± 1.4 and Bk_2_: 19% ± 3.0% from the baseline value; Figure 10V) and aortic vasodilation (Bk: 9 ± 1.6, Bk_1_: 14 ± 2.2 and Bk_2_: 19% ± 2.7% from the baseline value; Figure 10C). The association of Bk or Bk_2_ with BTCI (3 µg·kg^−1^, i.v.) did not change hypotension (Bk: −10 ± 1.1 and Bk_2_: −12 ± 2.7 mmHg from the baseline value; Figure 10A), but potentiated renal (Bk: 12 ± 1.1 and Bk_2_: 29% ± 4.9% the baseline value; Figure 10B) and aortic vasodilation (Bk: 16 ± 1.4 and Bk_2_: 28% ± 2.6% from the baseline value; Figure 10C). The association of Bk_1_ with BTCI did not alter hypotension (−10 ± 1.1 mmHg from the baseline value; Figure 10), renal (11% ± 1.4% from the baseline value; Figure 10) and aortic vasodilation responses (14% ± 2.0% from the baseline value; Figure 10).

### 2.8. Discussion

Bk and its analogues are peptides of pharmacological interest that can induce arteriolar dilation, nociceptor hypersensitivity and gastrointestinal smooth muscle contraction. Their degradation by different classes of proteases in plasma and tissues leads to a decrease in their half-life. So far, studies have focused on inhibiting metalloproteases and some serine proteases to increase Bk half-life or its release, respectively, in order to treat heart diseases and hereditary angioedema [9]. The present work describes biophysical characterization of two classes of biologically active molecules, the serine protease inhibitor BTCI and the peptide bradykinin and its analogues, Bk_1_ and Bk_2_. The hydrodynamic parameters of BTCI and Bk and its analogues, as well as the possible self-association tendency and complex formation between both molecules, were investigated. DLS measurement shows increased hydrodynamic diameter of BTCI in the presence of peptides indicating the formation of different complexes between BTCI and each Bk. These interactions are probably driven by hydrophobic forces, considering mainly the presence of phenylalanine amino acid residues in the Bk and valine present in the Bk analogues (Table 1) and the unusual hydrophobic surface of BTCI [28,29,32,33,34]. In addition, the BTCI-Bk complex formation was also indicated by the monodispersivity feature (polydispersity of 0.107) and the hydrodynamic diameter of about 3.3 nm, compatible with the dimensions of both associated molecules. It is intriguing the absence of the Bk and analogues light scattering signal upon complexes with BTCI. It is possible that the predominance of the light scattered signal of the complexes avoid the detection of the peptides.

Fluorescence quenching assays have been used as a tool to sense the solvent accessibility and the microenvironment of tryptophan residues, which are directly associated with conformational changes in proteins or protein-protein complex formation. BTCI presents one single tryptophan residue (Trp^82^) [26], which is largely exposed to the solvent and dominates the fluorescence signal of this protein, which is a rational strategy to monitor protein conformational changes and protein binding processes [41]. In this assay, fluorescence quenching was the primary structural parameter considered as a consequence of BTCI-Bk interactions.

The values of the Stern-Volmer constants (K_sv_), obtained as a function of pH, are two orders of magnitude larger than those for diffusion-limited quenching of free tryptophan in water. It can be attributed to static quenching process [41], caused by BTCI-Bk complex formation before the excitation state. It also shows that hydrophobic interactions between BTCI and Bk can occur near the tryptophan residue as well as within the hydrophobic environment of both molecules. In addition, it may also occur due to changes in ionization of amino acid residues surrounding Trp^82^ as a function of different pH condition, favoring the complex formation.

These results are in agreement with those obtained for BTCI-Bk binding assays, in which, comparatively, the higher affinity of Bk to BTCI occurred concomitantly, with conformational changes in the neutral (Bk and Bk_1_) and alkaline (Bk_2_) conditions. The number of binding sites (*n*) of all three Bk was close to one and similar for all evaluated pHs, whereas the binding constants, despite of low affinity, were different. However, comparatively, the affinity of Bk to BTCI was observed to be higher for Bk in pH 7.4 and Bk_2_ in pH 9.0 (Table 3). As also indicated by DLS assays, these weak interactions between Bk and BTCI may be driven by hydrophobic forces, considering mainly phenylalanine amino acid residues present in the Bk and valine present in the Bk analogues (Table 1) and the unusual hydrophobic surface of BTCI [28,29,32,33,34].

Taking all the results into consideration, BTCI seems to be more subject to conformational changes in neutral and alkaline conditions as a result of its residual charges at these different pHs and the binding process occurring by hydrophobic forces. Taken together, these results also reinforce the hypothesis that BTCI can be used as a Bk carrier in the blood system pathway in order to protect Bk against proteolytic attack by proteases and their release in target tissues. Additionally, our findings also indicate that BTCI could remain stable in some tissues to inhibit chymotrypsin or trypsin-like enzymes that cleave and inactivate bradykinin *in situ*. As expected, the kinin-inactivating pathway differs in different tissues and it is probably responsible to differences between results from two bioassays of bradykinin activities using both bradykinin-related peptides Bk_1_ and Bk_2_.

In agreement with those results obtained for BTCI-Bk binding assays using DLS and fluorescence quenching, quantitatively the FAR-UV CD spectra of BTCI and BTCI in the presence of the Bk-related peptides indicate that Bk induced a smaller alteration in the secondary structure of BTCI than related peptides. As shown, BTCI forms a complex with all three Bk, but with highest affinity for Bk (Table 3), compatible with minor conformational changes promoting few alterations in its secondary structure content (Table 4).

In order to analyze the relationship between conformational changes in BTCI and its inhibitory activity in the presence of Bk, Bk_1_ or Bk_2_, serine proteases enzymatic assays were performed in the presence of Bk-related peptides. In all conditions, the inhibitory activities of BTCI at concentrations in which it showed ~100% of protease inhibition were very similar. Therefore, the Bk-related peptides did not affect the properties of the reactive sites of BTCI, and they probably form complexes with BTCI in a different region of this molecule, driven by hydrophobic forces, as discussed. The maintenance of BTCI inhibitory activity even with the conformational changes caused by the Bk-related peptides could be explained due to the independence between the reactive sites for trypsin and chymotrypsin located in opposite and independent domains of the molecule [28,29].

The action of Bk on smooth muscle was evaluated when they were in complex with BTCI, using guinea pig ileum preparations, a well-characterized smooth-muscle contraction assay, sensitive to Bk. Different concentrations of Bk up to 10 µM were able to induce smooth-muscle contraction in a concentration-responsive manner. However, Bk_1_ and Bk_2_ were unable to exert their effects on muscle contraction, suggesting that further structural processing for interaction with B_2_ receptors or a higher dose than that used in the present assay are necessary. For example, an analogue of bradykinin from frog skin with a similar sequence to Bk_2_ (Pro^2^→hydroxyproline) induced smooth muscle contraction with a concentration 10 fold higher (100 µM) than Bk. The recovered muscle contraction action was consistent both with primary structure identity shared with mammalian Bk and the slight proline amino acid residue modification [42].

When pretreated with trypsin, Bk_1_ and Bk_2_ are cleaved on Arg^9^-Val^10^ and Arg^9^-Gln^10^, respectively, and induced smooth muscle contraction, in a concentration-responsive manner. Therefore, exposure of Arg^9^ at the C-terminal of both Bk was of fundamental importance for interaction with mammalian B_2_ receptors. As is known, Arg^1^, Pro^2^, Gly^4^, Phe^5^, Pro^7^, Phe^8^ and Arg^9^ are very important for mammalian smooth muscle activation [43]. Interestingly, the presence of Val^1^ in both analogues was responsible for an approximately 1.3-fold higher contraction force at 10 µM than in Bk, after activation by trypsin treatment (Figure 7). In contrast, Bk analogues pretreated with chymotrypsin were not able to induce smooth muscle contraction (data not shown), highlighting the effect of chymotrypsin, which may decrease Bk action and their half-life time, even without promoting its activation by hydrolysis of the two C-terminal residues. In this case, despite the presence of Val^1^, which enhanced the contraction force on Bk pretreated with trypsin, the cleavage on Phe^5^ and Phe^8^ led to the complete inactivation of both peptides.

The association between Bk, Bk_1_ or Bk_2_ with BTCI, in the presence of trypsin or chymotrypsin, was also evaluated. Bk was able to induce smooth muscle contraction in a concentration-responsive manner even in association with BTCI in the presence of serine proteases (Figure 8). BTCI was able to inhibit protease activity and did not alter Bk properties on smooth muscle. On the other hand, Bk_1_ and Bk_2_ did not show any effects in the presence of BTCI and serine proteases. As discussed, the cleavage of both Bk by trypsin and the prevention of chymotrypsin cleavage are fundamental to enhance smooth muscle contraction of these analogues. In the presence of BTCI, the catalytic activities of both serine proteases are inhibited and may be a strategy to retain the peptides in inactivated form until they are delivered to target plasma and tissues. These results indicated that BTCI may be an appropriate circumstantial carrier of Bk-related peptides as it did not show any alterations in its inhibitory properties, nor did it alters Bk properties on smooth muscle.

The cardiovascular effects of Bk and its related peptides in association with BTCI or vehicle were evaluated *in vivo* experiments. Our results are consistent with the data in the literature, which showed the vasodilation and hypotension responses to Bk [3,4]. Moreover, in the present study, we identify newly synthesized Bk_1_ and Bk_2_ with effective vascular relaxation and consequent hypotensive effect*s*. Additionally, the association of a protease inhibitor, BTCI potentiated aortic and renal vasodilation induced by Bk and Bk_2_ peptides, but these effects were not observed in the Bk_1_. The potentiating of BTCI on Bk_2_ but not on Bk_1_ hypotensive effect can be explained by the differences of the C-terminal between both Bk-related peptides. Thus, the negatively charged residue at the C-terminus of Bk_1_ could release more slowly the active Bk fragment.

Considered together, the results demonstrated that the Bk-related peptides, in special Bk_2_, induced reduction of vascular resistance and consequent hypotension. Therefore this work contributes to our understanding of the therapeutic potential of Bk-related peptides in the treatment of hypertension. Future studies are expected to elucidate the toxicological and the mechanism of action of these new peptides.

## 3. Experimental

### 3.1. Synthesis and Purification of Bk-related Peptides and Purification of the BTCI

The Bk-related peptides identified in Table 1 were synthesized by standard solid-phase method using the FMOC strategy. FMOC-amino acids and reagents for peptide synthesis were purchased from NovaSyn TGA (Novabiochem, San Diego, CA, USA). The synthesis was carried out through steps of deprotection of the FMOC group using 25% (v/v) 4-metylpiperidine in dimethylformamide (DMF). The peptide bond was formed using O-benzotriazole-N,N,N',N'-tetramethyluranium hexafluoro-phosphate (HBTU) in DMF at room temperature for 90 minutes. The complete desired Bk-related peptide bonded into resin was obtained after successive cycles of removing FMOC groups and coupling with the subsequent amino acids, as confirmed by Kaiser assay [44]. The peptide was subjected to cleavage from the resin by 95% (v/v) trifluoroacetic acid (TFA), 2.5% (v/v) triisopropylsilane, 2.5% (v/v) in Milli-Q water and precipitated using diisopropylether (−20 °C). The precipitate was collected by filtration, solubilized using 50% (v/v) acetonitrile (ACN) and then lyophilized. Bk-related peptides were purified by HPLC (Class LC-10VP from Shimadzu Corp., Kyoto, Japan) using a semi-preparative C_18_ Vydac 218 TP 510 (Hesperia, CA, USA) reverse phase column with a linear gradient (5%–95%) of ACN. The purity of the samples was analyzed by matrix assisted laser desorption ionization-time of flight mass spectrometry (MALDI-TOF/MS) using an UltraFlex III instrument (Bruker Daltonics, Bremen, Germany) under positive reflector mode with external calibration. Concentration of the synthetic Bk-related peptides was estimated (Equation (1)), as follows [45]:

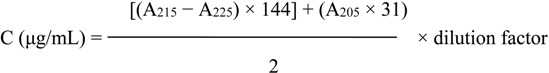
(1)

BTCI was purified as previously described [46] by ion exchange chromatography using DEAE-cellulose column (Sigma, St. Louis, MO, USA). The purity and molecular mass of BTCI were evaluated by MALDI-TOF/MS. BTCI concentration was determined using its specific absorption coefficient A_280_^1%^ = 8.23 and molecular mass of about 9.0 kDa.

### 3.2. Spectroscopic Measurements

#### 3.2.1. Dynamic Light Scattering (DLS) Assays

The hydrodynamic properties of the BTCI through the complex formation with Bk and analogues were monitored using DLS technique with laser source operated at 800 nm on the DynaPro LSR model (Wyatt Technology Corporation, Santa Barbara, CA, USA). The internal temperature was controlled by a Peltier system coupled to the detector. The Bk, Bk-related peptides, BTCI and BTCI-Bk, BTCI-Bk-related peptides complexes, were incubated for 30 minutes in 2 mM Tris-HCl pH 7.4 and then centrifuged at 5000 *g* and filtered (0.22 mm) before the DLS experiments. The average of 100 acquisitions for each measure was considered for BTCI (25 µM) and synthetic Bk-related peptides (25 µM) separately and subsequently for the BTCI-Bk complexes (1:1). The formation of complexes was analyzed at 37 °C, considering the relationship of the scattered light intensity signal and the hydrodynamic diameter of the samples, using the Dynamics V6 program (Wyatt Technology Corporation).

#### 3.2.2. Circular Dichroism (CD) Assays

Circular dichroism measurements were carried out using the Jasco J-815 spectropolarimeter (Jasco Analytical Instruments, Tokyo, Japan) equipped with a Peltier type temperature controller (Jasco Analytical Instruments). Far-UV spectra were recorded using a 0.1 cm path length quartz cuvette. After four consecutive measurements, signals were accumulated and the average spectrum was corrected for the baseline contribution of the buffer and Bk and Bk-related peptides solutions. Firstly, the CD spectra of BTCI (25 µM), Bk and Bk-related peptides (25 µM) were recorded independently in 2 mM Tris-HCl pH 7.4 at 37 °C. Subsequently, the CD spectrum of BTCI (25 µM) was recorded in the presence of all peptides (25 µM) in the same conditions. The secondary structure content of BTCI in the absence or presence of Bk and Bk-related peptides was estimated using the CDNN deconvolution software (Version 2.1).

#### 3.2.3. Fluorescence Spectroscopy Assays

Fluorescence measurements were performed at pH 5.0, 7.4 and 9.0 and 25 °C using the Jasco FP-6500 spectrofluorimeter (Jasco Analytical Instruments) coupled to a Jasco ETC-273T Peltier system (Jasco Analytical Instruments) with water circulation. Both excitation and emission slit were fitted to 5.0 nm and the excitation and emission wavelength were 295 nm and 300–400 nm, respectively. The concentration of BTCI was 25 µM, whereas the concentration of Bk and Bk-related peptides varied from 25 to 500 µM. The BTCI in complex with peptides were investigated by fluorescence quenching as a function of pH. The averages of five fluorescence spectra were recorded and processed by the program Spectra Manager (Jasco Analytical Instruments). The fluorescence emissions were fitted according to the classic Stern-Volmer equation (Equation (2)) [41]:

F_0_/F = 1 + K_SV_[Q] (2)

where F and F_0_ are the fluorescence intensities in the presence and absence of quencher, respectively, K_SV_ is Stern-Volmer constant and Q is the concentration of the quencher.

The binding constants for BTCI, Bk and Bk-related peptides interaction were calculated according to Equation (3):

Log[(F_0_ − F)/F] = LogK_b_ + *n*Log[Q] (3)

where K_b_ is the binding constant and *n* is the number of binding sites per BTCI [47].

### 3.3. Enzymatic Assay

The inhibitory activities of BTCI against bovine pancreatic trypsin (25 kDa; A_280_^1%^ = 15.90) and chymotrypsin (24 kDa; A_280_^1%^ = 20.40) (Sigma Co.) were first determined in the absence of Bk and Bk-related peptides [48]. Briefly, the residual enzymatic activities were assayed by mixing 40 µL of chymotrypsin (28.60 µM) or trypsin (2.57 µM) with BTCI at concentration range of 0–10 µM for trypsin and 0–40 µM for chymotrypsin at 25 °C for 15 minutes. After that, 200 µL of 0.40·mg·mL^−1^ N-glutaryl-L-phenylalanine-*p*-nitroanilide (GPNA) in 50 mM Tris-HCl, 20 mM CaCl_2_, pH 7.6 for chymotrypsin assay or 0.43 mg·mL^−1^ N_α_-benzoyl-DL-arginine 4-nitroanilide hydrochloride (BAPNA) in 50 mM Tris-HCl, 20 mM CaCl_2_, pH 8.2 for trypsin assay were added. Substrate hydrolysis was done for 15 minutes and stopped by adding 30% (v/v) acetic acid. The enzymatic assays were also carried out in the presence of Bk at similar concentration of BTCI. The relative enzymatic activities were evaluated by the liberation of *p*-nitroanilide, which was monitored at 410 nm using a SpectraMax Plus 384 Microplate Reader spectrophotometer (Molecular Devices, Sunnyvale, CA, USA). The obtained values are an average of three independent measurements. Inhibition curves were obtained by plotting relative activities of the proteases *vs.* BTCI concentration in the absence or presence of Bk and Bk-related peptides. Statistical analysis was carried out using the Paleontological Statistics (PAST) Software Package for Education and Data Analysis [49]. A value of Student’s *t*-test *p* < 0.05 was considered to denote a significant difference.

### 3.4. Smooth Muscle Constriction Force Assay using Mammalian Bk and Its Analogues Associated with BTCI

Male guinea pigs (*Cavia porcellus*) weighing between 200 and 300 g and fasted for 24 h were euthanized using an overdose of anesthetic at 120 mg/kg (ketamine and xylazine). The protocol was approved by the Committee on the Ethics of Animal Experiments of the Institute of Biology, University of Brasilia (Proc. Number 737402/2008). Segments of 2 cm from the terminal portion of the ileum were removed and washed in Tyrode’s solution (g/L: 8.00 NaCl,0.20 KCl, 0.20 CaCl_2_, 0.21 MgCl_2_), and attached to a glass rod that was immersed in a physiological bath chamber with a capacity of 10 mL containing Tyrode solution at 37 °C. In the opposite region, a thread of silk was attached to the F-60 isometric transducer connected to a polygraph (NARCO Bio-Systems, Houston, TX, USA). The bath was kept aerated constantly with carbogen (95% O_2_ + 5% CO_2_). Previously, as a control, smooth muscle response was analyzed using Bk from Sigma Aldrich. Experiments were done in triplicate, and constriction force was analyzed by addition of Bk and analogues in the absence or presence of BTCI, in three concentrations: 1, 5 or 10 µM. The hypotensive effects of the complexes were also evaluated in the presence of trypsin or chymotrypsin. After each experiment, the bath solution was exchanged three times in order to remove the drug used. Statistical analysis was carried out using the Paleontological Statistics (PAST) Software Package for Education and Data Analysis [49]. A value of Student’s *t*-test *p* < 0.05 was considered to denote a significant difference.

### 3.5. Evaluation of the Cardiovascular Effect of Intravenous Infusion of Bk, Bk_1_, Bk_2_ and BTCI

All experiments were conducted on adult male Wistar rats (280–350 g). They were obtained from the central animal house of the Federal University of Goiás. Experimental procedures were designed with strict adherence to the National Health Institute Guidelines for Care and Use of Laboratory Animals as approved by the Ethics Committee, Federal University of Goiás (034/12).

On the day of experiments, the rats were anesthetized with halothane (2%–3% in 100% O_2_; Cristália Ltda, Itapira, SP, Brazil) prior to the catheterization of the right femoral vein and artery. After the catheter placement, the rats were removed from the halothane and the anesthesia was maintained with urethane (1.2 g·kg^−1^ b.wt., i.v.; Sigma-Aldrich Co.). The trachea was cannulated to reduce airway resistance. The body temperature was kept at 37 ± 0.5 °C with a thermostatically controlled heated table.

Renal blood flow (RBF) and aortic blood flow (ABF) were measured with an ultrasonic transit-time flowmeter (Transonic Systems, Inc., Ithaca, NY, USA). Data were recorded continuously with an analog-to-digital converter (PowerLab 4/25, ML845, AD Instruments, Bella Vista, Australia). Mean arterial pressure (MAP) was determined from the pulsatile signal. Aortic vascular conductance (AVC) and renal vascular conductance (RVC) were calculated by a ratio of ABF and RBF divided by MAP, and were expressed as percentage of baseline. Increasing doses of BTCI (0.3, 3, 30 and 300 µg·kg^−1^, i.v.) were infused. In other group, the cardiovascular effects of Bk and its related peptides (Bk_1_ and Bk_2_,) at the dose of 120 µg·kg^−1^ (i.v.) in association with BTCI (3 µg·kg^−1^, i.v.) or vehicle (NaCl 0.9%) were tested.

Results are presented as means ± S.E.M. The effects of infusion of Bk, Bk-related peptides and BTCI on cardiovascular parameters were analyzed by one-way ANOVA. When groups differed significantly, the Fisher´s *post hoc* test was used by GraphPad Prism statistical software version 6.03 for Windows (GraphPad Software, Inc., San Diego, CA, USA). A value of Student’s *t*-test *p* < 0.05 was considered to denote a significant difference.

## 4. Conclusions

In the present study, we showed the actions of Bk and Bk-related peptides on smooth muscle in the presence of BTCI and also under the effect of the serine proteases trypsin and chymotrypsin, as well as *in vivo* effects of the peptides, isolated and associated with BTCI, in renal and aortic vascular reactivity. Our results indicate that formation of BTCI-Bk and BTCI-Bk-related peptides complexes is probably driven by hydrophobic forces. Moreover, no alteration in the inhibitory activity of BTCI in complex with any of the peptides was observed, and no alteration in smooth-muscle contraction properties of Bk occurred when associated to BTCI. However, when the BTCI-Bk complexes were tested *in vivo*, it was observed a potentiation renal and aortic vasodilation induced by Bk and Bk_2_ infusions. These results indicate that the BTCI-Bk-related peptides complexes may be a good strategy to act as a carrier and protective approach for the cleavage of serine proteases, leading to an increase in the half-life of Bk-related peptides. Additionally, our findings also indicate that BTCI could remain stable in some tissues to inhibit chymotrypsin or trypsin-like enzymes that cleave and inactivate bradykinin *in situ*. As expected, the kinin inactivating pathway differs in different tissues and it is probably responsible to differences between results from two bioassays of bradykinin activities using both bradykinin-related peptides Bk_1_ and Bk_2_. Furthermore, trials of nanoencapsulation of BTCI-Bk-related peptides could be performed to further evaluate its effects, and the half-life of Bk on a living system could be explored as a strategy to treat cardiovascular diseases.

## References

[1] Dobo J., Major B., Kekesi K.A., Szabo I., Megyeri M., Hajela K., Závodszky P., Gál P. (2011). Cleavage of kininogen and subsequent Bradykinin release by the complement component: Mannose-binding lectin-associated serine protease (MASP)-1. PLoS One.

[2] Mamenko M., Zaika O., Doris P.A., Pochynyuk O. (2012). Salt-dependent inhibition of epithelial Na^+^ channel—Mediated Sodium reabsorption in the aldosterone-sensitive distal nephron by bradykinin. Hypertens.

[3] Bhoola K.D., Figueroa C.D., Worthy K. (1992). Bioregulation of kinins: Kallikreins, kininogens, and kininases. Pharmacol. Rev..

[4] Regoli D., Gobeil F., Nguyen Q.T., Jukic D., Seoane P.R., Salvino J.M., Sawutz D.G. (1994). Bradykinin receptor types and B2 subtypes. Life Sci..

[5] Regoli D., Jukic D., Gobeil F., Rhaleb N.E. (1993). Receptors for bradykinin and related kinins: A critical analysis. Can. J. Physiol. Pharmacol..

[6] Carretero O.A., Scicli A.G. (1991). Local hormonal factors (intracrine, autocrine, and paracrine) in hypertension. Hypertension.

[7] McGiff J.C., Carroll M.A., Escalante B. (1991). Arachidonate metabolites and kinins in blood pressure regulation. Hypertension.

[8] Duka A., Duka I., Gao G., Shenouda S., Gavras I., Gavras H. (2006). Role of bradykinin B1 and B2 receptors in normal blood pressure regulation. Am. J. Physiol. Endocrinol. Metab..

[9] Golias C., Charalabopoulos A., Stagikas D., Charalabopoulos K., Batistatou A. (2007). The kinin system—Bradykinin: Biological effects and clinical implications. Multiple role of the kinin system—Bradykinin. Hippokratia.

[10] Potthast R., Ehler R., Scheving L.A., Sindic A., Schlatter E., Kuhn M. (2001). High salt intake increase uroguanylin expression in mouse kidney. Endocrinology.

[11] Carrithers S.L., Taylor B., Cai W.Y., Johnson B.R., Otto C.E., Gremberg R.N., Jackson B.A. (2000). Guanylyl cyclase-C receptor mRNA distribution along the rat nephron. Regul. Pep..

[12] Erdös E.G., Fulong T.F., Skidgel R.A. (2010). Angiotensin I-Converting enzyme inhibitors are allosteric enhancers of kinin B1 and B_2_ receptor function. Hypertension.

[13] Kumar R., Yong Q.C., Thomas C.M., Baker K.M. (2012). Intracardiac intracellular angiotensin system in diabetes. Am. J. Physiol. Regul. Integr. Comp. Physiol..

[14] Laskowski M., Qasim M.A. (2000). What can the structures of enzyme-inhibitor complexes tell us about the structures of enzyme substrate complexes?. Biochem. Biophys. Acta.

[15] Ryan C.A. (1991). Proteinase inhibitors in plants: Genes for improving defenses against insects and pathogens. Ann. Rev. Phytol..

[16] Franco O.L., Santos R.C., Batista J.A.N., Mendes A.C.M., Araújo M.A.M., Monnerat R.G., Grossi-de-Sá R.F., Freitas S.M. (2003). Effects of black-eyed pea trypsin/chymotrypsin inhibitor on proteolytic activity and on development of *Anthonomus grandis*. Phytochemistry.

[17] Tian M., Benedetti B., Kamoun S. (2005). A second kazal-like protease inhibitor from *Phytophthora infestans* inhibits and interacts with the apoplastic pathogenesis-related protease P69B of tomato. Plant Physiol..

[18] Kataoka H., Itoh H., Koono M. (2002). Emerging multifunctional aspects of cellular serine proteinase inhibitors in tumor progression and tissue regeneration. Pathol. Int..

[19] Esse H.P., Klooster J.W., Bolton M.D., Yadeta K.A., Baarlen P., Boeren S., Vervoort J., de Wit P.J.G.M., Thommas B.P.H.J. (2008). The *Cladosporium fulvum* virulence protein Avr2 inhibits host proteases required for basal defense. Plant Cell.

[20] Kennedy A.R. (1998). Chemopreventive agents: Protease inhibitors. Pharmacol. Ther..

[21] Armstrong W.B., Kennedy A.R., Wan X.S., Taylor T.H., Nguyen T.A., Jensen J., Thompsom W., Lagerberg W., Meyskens F.L. (2000). Clinical modulation of oral leukoplakia and protease activity by Bowman-Birk inhibitor concentrate in a phase IIa chemoprevention trial. Clin. Cancer Res..

[22] Kennedy A.R., Wan X.S. (2002). Effects of the Bowman-Birk inhibitor on growth, invasion, and clonogenic survival of human prostate epithelial cells and prostate cancer cells. Prostate.

[23] Chen Y.W., Huang S.C., Lin-Shiau S.Y., Lin J.K. (2005). Bowman-Birk inhibitor abates proteasome function and suppresses the proliferation of MCF7 breast cancer cells through accumulation of MAP kinase phosphatase-1. Carcinogenesis.

[24] Saito T., Sato H., Virgona N., Hagiwara H., Kashiwagi K., Suzuki K., Asano R., Yano T. (2007). Negative growth control of osteosarcoma cell by Bowman-Birk protease inhibitor from soybean; involvement of connexin. Cancer Lett..

[25] Joanitti G.A., Azevedo R.B., Freitas S.M. (2010). Apoptosis and lysosome membrane permeabilization induction on breast cancer cells by an anticarcinogenic Bowman-Birk protease inhibitor from *Vigna unguiculata* seeds. Cancer Lett..

[26] Morhy L., Ventura M.M. (1987). The complete amino acid sequence of the *Vigna unguiculata* (L.) WaLP seed trypsin and chymotrypsin inhibitor. An. Acad. Bras. Cienc..

[27] Ventura M.M., Martin C.O., Morhy L. (1975). A trypsin and chymotrypsin inhibitor form black-eyed pea (*Vigna sinensis* L.). VI. Isolation and properties of complexes with trypsin and chymotrypsin. An. Acad. Bras. Cienc..

[28] Freitas S.M., Mello L.V., Silva M.C., Vriend G., Neshich G., Ventura M.M. (1997). Analysis of the black-eyed pea trypsin and chymotrypsin inhibitor-α-chymotrypsin complex. FEBS Lett..

[29] Barbosa J.A.R.G., Silva L.P., Teles R.C.L., Esteves G.F., Azevedo R.B., Ventura M.M., Freitas S.M. (2007). Crystal structure of the Bowman-Birk inhibitor from *Vigna unguiculata* seeds in complex with β-Trypsin at 1.55 Å resolution and its structural properties in association with proteinases. Biophys. J..

[30] Carvalho A.F., Santos-Neto M.S., Monteiro H.S.A., Freitas S.M., Morhy L., Nascimento N.R.F., Fonteles M.C. (2008). BTCI enhances guanylin-induced natriuresis and promotes renal glomerular and tubular effects. Braz. J. Biol..

[31] Borgstahl G.E.O., Doublié S. (2007). How to use Dynamic Light Scattering to improve the likelihood of growing macromolecular crystals. Macromolecular Crystallography Protocols. Methods in Molecular Biology.

[32] Ventura M.M., Mizuta K., Ikemoto H. (1981). Self-association of the black-eyed pea trypsin and chymotrypsin inhibitor in solution. A study by light scattering. An. Acad. Bras. Cienc..

[33] Silva L.P., Azevedo R.B., Morais P.C., Ventura M.M., Freitas S.M. (2005). Oligomerization states of Bowman-Birk inhibitor by atomic force microscopy and computational approaches. Prot. Struct. Funct. Bioinform..

[34] Ventura M.M., Mizuta K., Ikemoto H. (1984). Solvent perturbation and surface accessibility of the tryptophyl and tyrosyl groups in black-eyed pea trypsin and chymotrypsin inhibitor. An. Acad. Bras. Cienc..

[35] Brady A.H., Ryan J.W. (1971). Circular Dichroism of Bradykinin and Related Peptides. Biochem. J..

[36] Cann J.R., Stewart J.M., Matsuedai G.R. (1973). A circular dichroism study of the secondary structure of Bradykinin. Biochemistry.

[37] Kotovych G., Cann J.R., Stewart J.M., Yamamoto H. (1998). NMR and CD conformational studies of bradykinin and its agonists and antagonists: Application to receptor binding. Biochem. Cell Biol..

[38] Fachetti H.C.S., Mizuta K., Ventura M.M. (1984). Thermodynamics of the black-eyed pea trypsin and chymotrypsin inhibitor. An. Acad. Bras. Cienc..

[39] Freitas S.M., Ikemoto H., Ventura M.M. (1999). Thermodynamics of the binding of chymotrypsin with the black-eyed pea trypsin and chymotrypsin inhibitor (BTCI). J. Protein Chem..

[40] Böhm G., Muhr R., Jaenicke R. (1992). Quantitative analysis of protein far UV circular dichroism spectra. Protein Eng..

[41] Lakowicz J.R. (2006). Principles of Fluorescence Spectroscopy.

[42] Brand G.D., Krause F.C., Silva L.P., Leite J.R.S.A., Melo J.A.T., Prates M.V., Pesquero J.B., Santos E.L., Nakaie C.R., Costa-Neto C.M. (2006). Bradykinin-related peptides from *Phyllomedusa hypochondrials*. Peptides.

[43] Regoli D., Barabé J. (1980). Pharmacology of bradykinin and related kinins. Pharmacol. Rev..

[44] Kaiser E., Colescott R.L., Bossinger C.D., Cook P. (1970). Color test for detection of free terminal amino groups in the solid phase synthesis of peptides. Anal. Biochem..

[45] Murphy J.B., Kies M.W. (1960). A note on spectrophotometric determination of proteins in dilute solution. Biochim. Biophys. Acta.

[46] Ventura M.M., Xavier-Filho J. (1996). A trypsin and chymotrypsin inhibitor from black-eyed pea (*Vigna sinensis*). I. Purification and partial characterization. An. Acad. Bras. Cienc..

[47] Hu Y.J., Liu Y., Hou A.X., Zhao R.M., Qu X.S., Qu S.S. (2004). Studies on the interaction between rare-earth salts of heteropoly EuHSiMo_10_-W_2_O_40_·25H_2_O and bovine serum albumin. Acta Chim. Sin..

[48] Erlanger B.F., Kokowsky N., Cohen W. (1961). The preparation and properties of two new chromogenic substrates of trypsin. Arch. Biochem. Biophys..

[49] Hammer M., Harper D.A.T., Ryan P.D. (2001). PAST: Paleontological Statistics Software Package for Education and Data Analysis. Palaeontol. Electron..

